# An Olympic Skier Returning to Competition After a Complex Knee Reconstruction: A Case Report

**DOI:** 10.7759/cureus.71879

**Published:** 2024-10-19

**Authors:** Peter Verdonk, Rene Verdonk, Sacha Beca

**Affiliations:** 1 Orthopaedics, AZ Monica, Antwerp, BEL; 2 Orthopedic Surgery, Hôpital Erasme, Brussels, BEL; 3 Family Medicine, Université Libre de Bruxelles, Brussels, BEL

**Keywords:** acl, ligament injuries, meniscal injuries, mlki, professional athlete, ski

## Abstract

Alpine skiing is a high-risk sport due to the possibility of severe injuries, particularly complex knee injuries. The most common injuries are ruptures of the anterior cruciate ligament (ACL), meniscal tears and fractures of the lower limbs. Managing these injuries requires ligament reconstructions and specific surgical interventions to optimize rehabilitation and ensure a return to competition. This case report describes the incident of a young Olympic skier who sustained a severe knee injury following a fall during training. The result was a fracture of the left posteromedial tibial plateau, a detachment of the medial collateral ligament, an ACL rupture, and tears of both menisci. Management involved a total of seven surgical procedures and months of intensive physiotherapy. ACL reconstruction was performed using an autologous patellar tendon graft. It took three years before the patient was able to come back to competition.

ACL reconstruction and rehabilitation require varying recovery periods depending on the injury, with a possible return to practice after 4 to 12 months. Meniscal and multi-ligament injuries demand personalized protocols and precise management to optimize recovery. This case report highlights the complex challenges associated with knee surgery and rehabilitation in the context of sports injuries. The management of multiple ligament and meniscus injuries requires a sophisticated surgical approach, including the use of advanced grafts and techniques. Post-operative rehabilitation is equally crucial, requiring customized protocols to optimize recovery. This case illustrates the importance of integrated, rigorous management to enable a person's safe return to the sporting activity, while minimizing the risk of recurrence.

## Introduction

History of skiing and injuries

Alpine skiing has been recognized as an Olympic sport since the 1960s. It includes four disciplines: downhill, super-giant, giant slalom, and slalom. The slalom, considered the most technical event, is characterized by shorter gate distances, tighter turns, slower speeds, and less steep slopes. However, skiers can still reach high speeds, and environmental factors, such as the slope’s gradient, can influence performance [1]. These characteristics make alpine skiing a high-risk sport. Injuries are frequently reported, with an incidence ranging from 23.5 to 36.7 injuries per 100 athletes per season at the World Cup level. Approximately 45% of injuries occur during competition rather than training [2]. Because skiing puts so much strain on the lower limbs, the knee is the most frequently injured area among adult alpine skiers. Anterior cruciate ligament (ACL) injuries are the most common diagnosis, followed by concussions and lower limb fractures. ACL tears primarily occur in situations involving loss of balance, awkward landings, or falls [2,3]. In elite athletes, ACL injuries can be severe and have a significant impact on performance. Reconstruction is crucial to restore knee stability and athletic function.

Multi-ligament knee injuries

Among skiers who have suffered ACL tears, associated knee injuries are common. A study of surgical reports of 28 elite alpine skiers with ACL injuries revealed that only 18% had isolated ACL tears; 32% had multi-ligament knee injuries (MLKI), 54% presented cartilage damage, and 61% sustained meniscal injuries [4].

MLKI are severe injuries defined as damage involving at least two of the four major knee ligaments: the anterior cruciate ligament, the posterior cruciate ligament (PCL), the lateral collateral ligament, or LCL (including the posterolateral corner), and the medial collateral ligament (MCL) [5]. It is important to note that MLKI are strongly associated with meniscal tears and cartilage lesions, with combined rates ranging from 27% to 30%. Additionally, between 11% and 19% of MLKI cases are associated with peroneal nerve injuries, vascular injuries, and articular fibrosis [6].

Meniscal tears

Meniscal injuries are commonly encountered in athletes and often require surgical intervention through arthroscopy. Common surgical approaches for treating meniscal tears include partial meniscectomy and meniscal repair. Meniscal tears are classified based on their type and location [7,8]. The most crucial variable for an athlete with a meniscal injury is the post-operative timeline for returning to the sports activity. For the surgeon, the challenge is to balance the urgency of returning to play while preserving knee function, performance, and long-term functional longevity [8].

Magnetic resonance imaging (MRI) plays a crucial role in diagnosing meniscal injuries. Various types exist, with descriptions differing across practices and disciplines. Due to the lack of consistent terminology and the heterogeneity of classifications in the literature, the International Society of Arthroscopy, Knee Surgery, and Orthopaedic Sports Medicine (ISAKOS) has recommended and validated a classification system to characterize meniscal tears in arthroscopy and enhance the reliability of injury assessment and documentation [9].

Ligament reconstruction and grafts

Many treatment options are available for knee ligament injuries. For example, the initial treatment of an ACL sprain includes analgesics, arthrocentesis and, if necessary, the use of a rigid splint. Surgery may be proposed as a curative measure if the patient complains of excessive anterior knee instability, or as a preventive measure to preserve the menisci and avoid premature knee osteoarthritis. ACL reconstruction may then be proposed by ligamentoplasty.

Commonly used autografts include hamstring tendon (HT), bone-patellar tendon-bone (BPTB), and quadriceps tendon (QT). While HT and BPTB have traditionally been favored, the QT graft has gained increasing interest in recent years due to its clinical stability and comparable risk profile to other graft types [10-12].

## Case presentation

**Figure 1:**
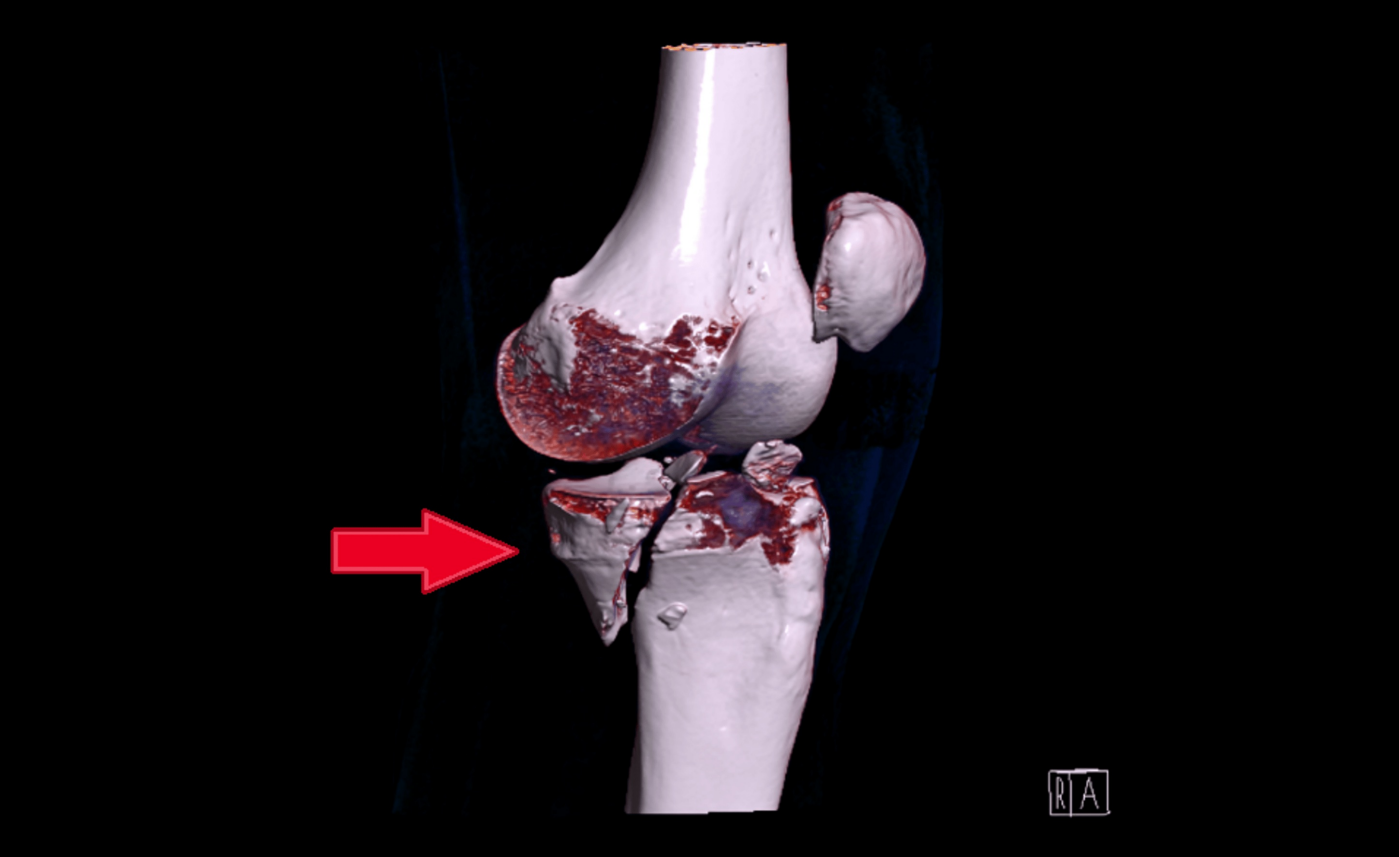
A CT scan showing a fracture of the left posteromedial tibial plateau, with the volume rendering technique (anteromedial view of the left knee)

**Figure 2:**
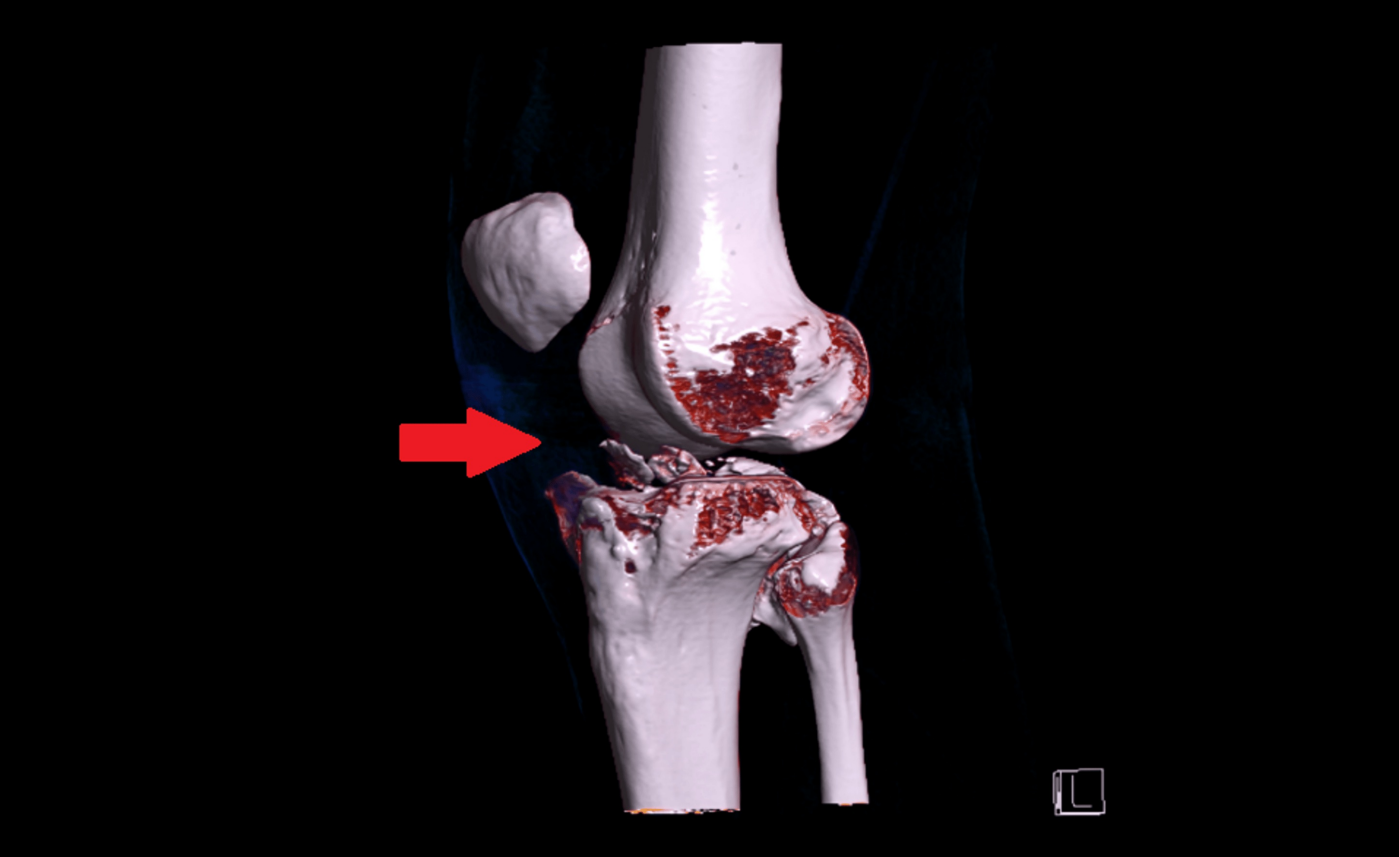
A CT scan showing the fracture with the volume rendering technique (anterolateral view of the left knee)

**Figure 3:**
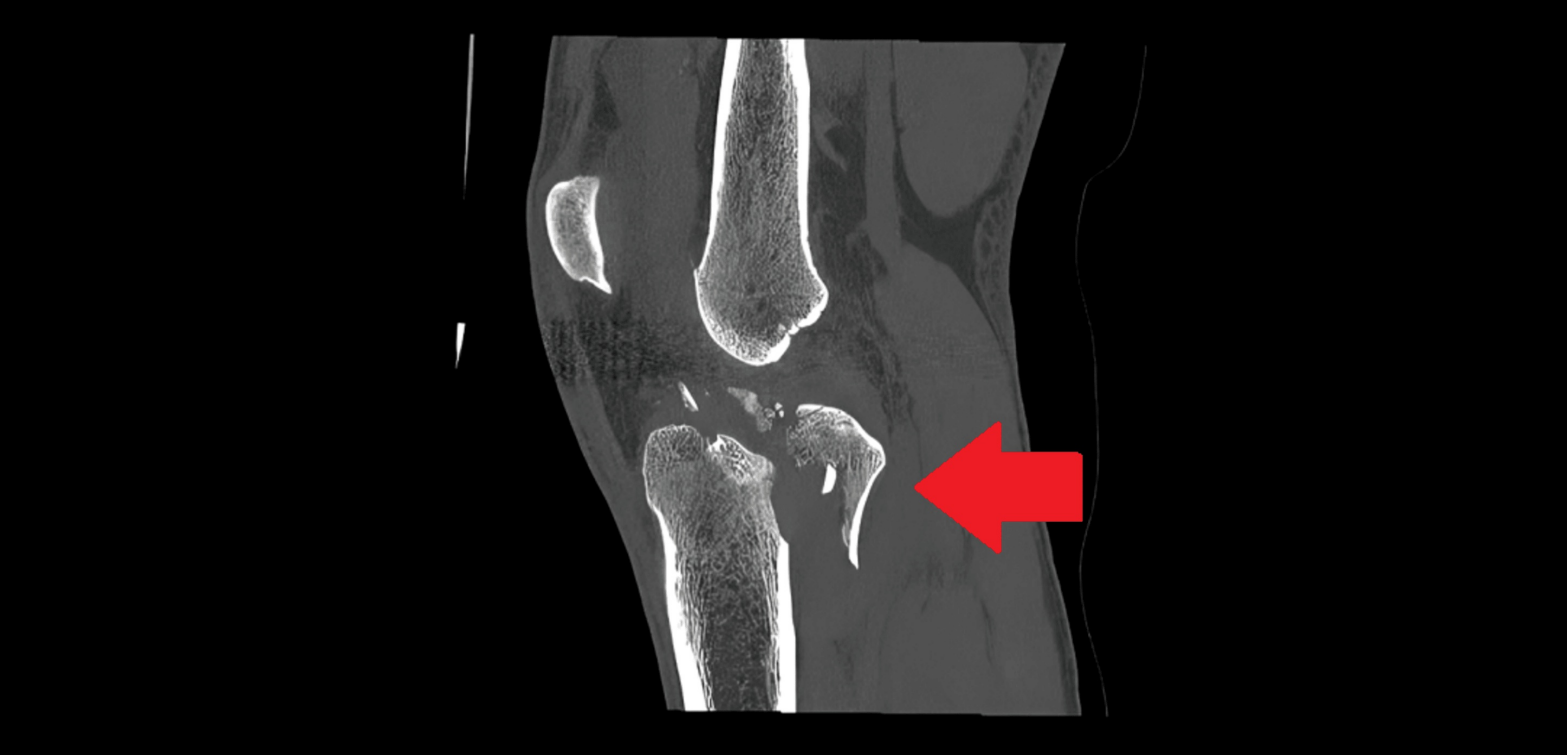
A CT scan showing the fracture, with sagittal view of the left knee

The patient was a young Olympic skier who sustained a severe fall during a giant slalom training session in January 2017. The result was a fracture of the left posteromedial tibial plateau, a detachment of the medial collateral ligament, an anterior cruciate ligament rupture, and tears of both menisci. Figures 1-3 are CT scan images showing a fracture of the posteromedial tibial plateau following the fall during training, with 3D reconstructions using direct volume rendering technology (Figures 1-2).

**Figure 4:**
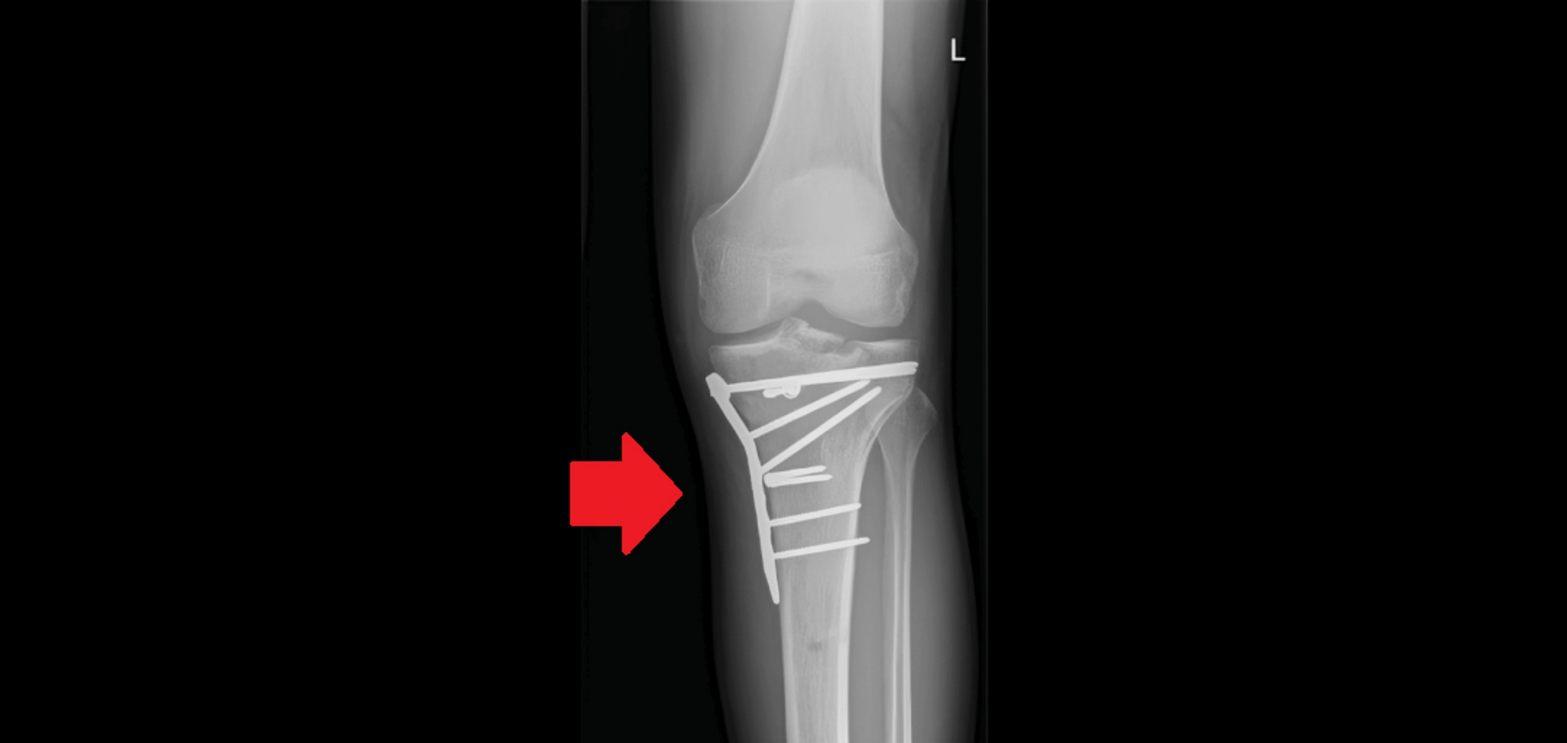
Radiography showing open reduction and internal fixation (ORIF) of the tibial plateau with the placement of an anteroposterior screw (anteroposterior view of the left knee)

The initial intervention, which took place five days after injury, included an open reduction and internal fixation (ORIF) of the tibial plateau with placement of an anteroposterior screw, medial meniscus lateral repair, repair of the medial collateral ligament with staples, and suturing of the anterior cruciate ligament to the intercondylar eminence. At this stage, it was discussed with the patient that a return to competition was generally only possible after 9 to 12 months of rehabilitation. Figure 4 shows the post-operative result after ORIF surgery.

Between February and August 2017, a progressive loss of joint mobility was observed. An arthroscopy performed in April revealed significant articular fibrosis. Increased laxity of the anterior cruciate ligament necessitated a further operation in the month of September in the same year. Surgery consisted of ACL reconstruction using a 10-cm graft from the autologous patellar tendon, repair of the anterolateral ligament, and extra-articular stabilization with a fascia lata graft. By the end of 2017, rehabilitation was progressing well, with clinical improvement and knee stability. A return to competition was initially anticipated for May 2018.

In February 2018, an arthroscopy showed a free fragment from the anterior horn of the medial meniscus as well as the presence of micro-fractures. A follow-up indicated good healing and improved stability, allowing continued rehabilitation. However, persistent pain, discomfort, and inability to resume sports led to two additional surgeries later in the year. The first surgery involved removal of the osteosynthesis material. The second surgery addressed the removal of the suture from the anterior horn of the medial meniscus, resection of the medial posterior horn, and release of the knee extensor mechanism through arthrolysis. In December 2018, the patient was able to ski again for the first time since the accident, and at the end of 2019, he returned to competition.

Between 2019 and 2020, the patient reported minimal complaints, and clinical exams showed joint stability. Rehabilitation continued, with a few autologous protein solution injections. In 2021, the patient experienced recurrent infrapatellar pain in the right knee, requiring the resection of an osteophyte through arthroscopy. This procedure also demonstrated normalization of intra-articular lesions. Ostenil injections were regularly administered to manage inflammation and improve joint function.

From 2022 to 2024, the management focused on addressing anterior pain with injections of hyaluronic acid, and consideration for resection of bothersome osteophytes. In conclusion, the patient underwent seven surgeries before he was able to return to competition three years after his injury. Table 1 provides a detailed summary of all surgeries performed over a four-year period.

**Table 1:** Summary of all surgical procedures performed ACL, anterior cruciate ligament

Date	Procedures
January 2017	Left knee surgery: open reduction and internal fixation with placement of an anteroposterior screw; medial meniscus suture; fixation of the medial collateral ligament; ACL fixation on the intercondylar eminence
April 2017	Left knee arthroscopy: status post-ACL rupture; status post-medial meniscus suture; articular fibrosis
September 2017	Left knee arthroscopy: status post-ACL rupture with laxity; status post-medial meniscus suture with partial tear of the inner rim
	Left knee surgery: ACL reconstruction using a 100-mm autologous patellar graft; graft fixation with an 8x25 mm RCI screw; extra-articular stabilization with a fascia lata graft (Monoloop procedure)
February 2018	Left knee arthroscopy: micro-fractures; free fragment in the anterior horn of the medial meniscus; good fixation of the meniscal horn
June 2018	Left knee surgery: removal of the osteosynthesis material
September 2018	Left knee arthroscopy: presence of adhesions in the lateral compartment and femoropatellar joint
	Left knee surgery: removal of the anterior horn meniscus medial lateral suture; partial resection of the posterior horn of the medial lateral meniscus; release of the extensor apparatus through arthrolysis and relaxation of the quadriceps
April 2021	Right knee arthroscopy: intact intra-articular compartments
	Right knee surgery: mini-open resection of the osteophyte at the distal patellar pole of the right knee

## Discussion

ACL injuries and return to high-level sports

When it comes to sports injuries, a surgeon's main objective is to restore the athlete's optimal performance level by re-establishing knee stability and function. This involves rigorous rehabilitation and appropriate post-operative care. The post-operative timeline for returning to sports varies by injury type: 9 to 12 months for multi-ligament injuries, 7 to 9 months for isolated ACL reconstruction, and 4 to 6 months for isolated meniscal repair [13]. Although ACL injuries are common among skiers, the return-to-sport (RTP) rate remains high because alpine skiing does not require intense directional changes or pivoting movements [14].

Despite the low demand placed on the ACL in alpine skiing, effective rehabilitation using various therapies is crucial for ensuring a safe return to the sport after reconstruction. According to Glattke et al., supervised rehabilitation, neuromuscular electrical stimulation, and psychological readiness assessment are essential for a secure return to sport after ACL reconstruction [15]. The surgeon is responsible for determining the appropriate time for the athlete to resume sports, ensuring that the post-operative period is sufficient and that the healing of the injuries is optimal. Returning to a sport that stresses the knee less than nine months after ACL reconstruction increases the risk of a second ACL injury by a factor of seven [16].

Meniscal injuries

Many studies have been conducted to analyze the effects of total meniscectomy and compare it with less radical techniques such as radial meniscal repair. The current literature indicates that radial repair tends to produce more favorable results, due to growing concerns about long-term arthritis following meniscectomy. Additionally, meniscectomy is a significant predictor of the inability to return to sports [17,18]. It is not possible to apply a standardized protocol to precisely assess an athlete's return to high-level performance after a meniscal injury. Two athletes with a meniscal injury will not follow the same rehabilitation path to achieve full recovery of their performance. A multidisciplinary approach to care is necessary and should be applied to all patients [8].

According to Calanna et al., despite the numerous protocols available in the literature, there is no consensus on a specific rehabilitation program or the timeline for resuming sports activity after an isolated meniscal repair. Biomechanical data suggest that tailoring a personalized protocol based on the type of injury and meniscal stability is a reasonable approach [19].

Gastaldo et al. highlighted the importance of precisely controlling the progressive increase in loads, maintaining frequent medical consultations, and conducting detailed functional tests on the quantity and quality of movements for athletes with meniscal injuries. Additionally, monitoring the return to sport should be comprehensive and interdisciplinary, incorporating state-of-the-art tests to achieve optimal physical condition and effective progression in field rehabilitation [20].

There is a very limited number of case reports concerning skiers who have suffered from meniscal and multi-ligament injuries. The scarcity of sport-specific studies prevents a thorough understanding of the particularities of these injuries in skiing. The lack of detailed data limits the development of treatment and rehabilitation protocols tailored to the unique demands of alpine skiing, highlighting a crucial need for further research and documentation on these specific cases.

Management of MLKI

As shown in Table 2, the Schenck classification system has been developed to categorize knee dislocations based on multi-ligament knee injuries [21].

**Table 2:** Schenck classification MLKI, multi-ligament knee injuries; KD, knee dislocation; ACL, anterior cruciate ligament; PCL, posterior cruciate ligament; MCL, medial collateral ligament; LCL, lateral collateral ligament; PLC, posterolateral corner; M, medial; L, lateral

Type	Characteristics
KD I	MLKI with either ACL or PCL injury
KD II	MLKI with ACL and PCL injury
KD III-M	MLKI with ACL and PCL and MCL injury
KD III-L	MLKI with ACL and PCL and LCL and PLC injury
KD IV	MLKI with panligament rupture
KD V	Knee fracture-dislocation

Ng et al. synthesized key points in the management of multi-ligament knee injuries based on the best available evidence. Immediate care is essential to identify and address any potential vascular and nerve injuries, as 18% of MLKI cases are associated with vascular injuries. Surgical treatment, whether performed acutely or in delayed stages over several weeks, yields the best results. Finally, early mobilization leads to reduced range of motion deficits [22].

According to a study conducted by Bakshi et al., involving National Football League players, the RTP rate for athletes with multi-ligament knee injuries is significantly lower than for those with isolated ACL tears. Additionally, athletes with ACL and MCL tears have a higher RTP rate, a significantly shorter RTP timeline, and a greater likelihood of returning to their previous level of performance compared to those with ACL and PCL/LCL tears [23].

Everhart et al. conducted a systematic review revealing that approximately 60% of patients who undergo surgery for multi-ligament knee injuries return to sports, though returning to high-level sports is less common. Returning to work is often feasible after a multi-ligament injury, but may require adjustments in tasks or job position. Additionally, factors such as obesity, non-operative treatments, increased injury severity, and vascular injuries are associated with less favorable functional outcomes [24].

Graft fixation techniques

Dave et al. demonstrated in a systematic review that autografts from the patellar tendon and quadriceps tendon provide similar improvements in knee stability and functional outcomes post-ACL reconstruction, with no significant differences in complications [25]. In a meta-analysis conducted by Mouarbes et al., it was found that the quadriceps tendon graft resulted in less pain at the donor site compared to the patellar tendon graft and provided better functional outcomes than the hamstring tendon graft [26]. Widner et al. demonstrated that irradiated allografts present an increased risk of failure in younger athletes and should therefore be reserved for revision cases and those aged 35 and over. Ultimately, the choice of graft should be based on the surgeon's comfort and experience, as well as the patient's individual characteristics [27].

Jackson et al. noted that complications after primary ACL reconstruction with an autologous quadriceps graft occurred in 10.5% of cases, with anterior knee pain being the most common. Although the overall incidence of complications was similar between QT and BPTB grafts, anterior pain was 2.7 times more common with the soft-tissue quadriceps graft [28].

Graft fixation techniques, whether through soft tissue or bone, are crucial considerations in ligament reconstruction. Although some techniques might offer biomechanical advantages, there is no consensus on their clinical superiority. Further research is needed to assess their long-term impact, particularly in high-level athletes.

In summary, there are various techniques for knee ligament reconstruction, with different graft types offering distinct advantages and disadvantages. Table 3 provides a comparative overview of the commonly used grafts [29].

**Table 3:** Comparative overview of graft types ACL, anterior cruciate ligament; PCL, posterior cruciate ligament; SMCL, superficial medial collateral ligament; PMC, posteromedial corner; PLC, posterolateral corner

Type	Reconstruction	Advantages	Disadvantages
Bone-patellar tendon-bone (BPTB)	ACL/PCL	Thick and robust graft	Knee pain; patellar fracture; patellar tendon rupture; surgical duration
Hamstring tendon (HT)	ACL/PCL/PLC/PMC/SMCL	Long graft; easy to harvest; low donor site morbidity	Tissue adhesions; availability; surgical duration
Quadriceps tendon (QT)	ACL/PCL	Thick and robust graft; low donor site morbidity	Knee pain; patellar fracture; patellar tendon rupture; surgical duration

Limitations

The limitations inherent in a case report are present in this work. This is a study of a single individual, which means that the results cannot be extrapolated to the general population or to athletes undergoing similar procedures. Although this athlete was able to resume his sporting activity, these results cannot be generalized to other sports or levels of competition. Furthermore, the specificity of the injury pattern and the unique clinical context may limit the overall interpretation of these results.

## Conclusions

This case report highlights the complex challenges associated with knee surgery and rehabilitation in the context of sports injuries. The management of multiple ligament and meniscus injuries requires a sophisticated surgical approach, including the use of advanced grafts and techniques. Post-operative rehabilitation is equally crucial, requiring customized protocols to optimize recovery. This case illustrates the importance of integrated, rigorous management to enable a safe return to sporting activity, while minimizing the risk of recurrence.
